# Identification and Characterization of Bacterial Vaginosis-Associated Pathogens Using a Comprehensive Cervical-Vaginal Epithelial Coculture Assay

**DOI:** 10.1371/journal.pone.0050106

**Published:** 2012-11-15

**Authors:** Colleen R. Eade, Camila Diaz, Matthew P. Wood, Kathryn Anastos, Bruce K. Patterson, Phalguni Gupta, Amy L. Cole, Alexander M. Cole

**Affiliations:** 1 Department of Molecular Biology and Microbiology, Burnett School of Biomedical Sciences, College of Medicine, University of Central Florida, Orlando, Florida, United States of America; 2 Departments of Medicine and Epidemiology and Population Health, Albert Einstein College of Medicine, Bronx, New York, United States of America; 3 Department of Pathology, Division of Infectious Diseases and Geographic Medicine, Stanford University School of Medicine, Palo Alto, California, United States of America; 4 Department of Infectious Diseases and Microbiology, Graduate School of Public Health, University of Pittsburgh, Pittsburgh, Pennsylvania, United States of America; Columbia University, United States of America

## Abstract

Bacterial vaginosis (BV) is the most commonly treated female reproductive tract affliction, characterized by the displacement of healthy lactobacilli by an overgrowth of pathogenic bacteria. BV can contribute to pathogenic inflammation, preterm birth, and susceptibility to sexually transmitted infections. As the bacteria responsible for BV pathogenicity and their interactions with host immunity are not understood, we sought to evaluate the effects of BV-associated bacteria on reproductive epithelia. Here we have characterized the interaction between BV-associated bacteria and the female reproductive tract by measuring cytokine and defensin induction in three types of FRT epithelial cells following bacterial inoculation. Four BV-associated bacteria were evaluated alongside six lactobacilli for a comparative assessment. While responses differed between epithelial cell types, our model showed good agreement with clinical BV trends. We observed a distinct cytokine and human β-defensin 2 response to BV-associated bacteria, especially *Atopobium vaginae*, compared to most lactobacilli. One lactobacillus species, *Lactobacillus vaginalis*, induced an immune response similar to that elicited by BV-associated bacteria, stimulating significantly higher levels of cytokines and human β-defensin 2 than other lactobacilli. These data provide an important prioritization of BV-associated bacteria and support further characterization of reproductive bacteria and their interactions with host epithelia. Additionally, they demonstrate the distinct immune response potentials of epithelial cells from different locations along the female reproductive tract.

## Introduction

Bacterial vaginosis (BV) is the most common disorder of the female reproductive tract (FRT) for which clinical intervention is sought [1]. BV is a microbial shift condition, characterized by the displacement of commensal vaginal lactobacilli and the overgrowth of mixed pathogenic bacterial populations [2]. Neither the presence nor absence of any single bacterial species is sufficient for diagnosis, but instead multifactorial clinical and microbiological criteria are used to diagnose BV [3], [4]. This condition affects between 20–60% of women worldwide, and can pose serious immediate and long-term sequelae [5], [6], [7]. Women who have BV are at a higher risk of developing pelvic inflammatory disease, and pregnant women experiencing BV are significantly more likely to encounter complications, including preterm birth [8]. Furthermore, BV increases a woman’s chance of acquiring sexually transmitted infections, including HIV, whose acquisition rate is increased by 60% in women experiencing BV [6]. These serious clinical consequences of BV, combined with its high prevalence, make this condition of immediate priority.

Research has just begun to elucidate the mechanisms of BV pathogenicity. It is apparent that bacterial pathogens elicit an immune response in the FRT. This response is characterized by an upregulation of proinflammatory cytokines, such as IL-8, IL-1α, and IL-1β, yet the host cells responsible for pathogen recognition and immune response remain uncharacterized [9], [10], [11], [12]. Other aspects of the host innate immune response to BV are even less clear; there is conflicting evidence regarding regulation of antimicrobial effector proteins, such as the human β-defensins (hBDs). This family includes antibacterial peptides that are reported to be induced by bacterial stimuli [13], yet studies of hBD regulation in the context of BV have had conflicting results, with some studies showing a significant increase in hBD levels in the FRT coincident with BV, and others reporting a significant decrease [14], [15]. Furthermore, with dozens of bacterial species associated with the microbial shift that defines BV, researchers have yet to characterize the pool of candidate pathogens and elucidate their immunostimulatory properties [16], [17], [18], [19].

With insufficient characterization of pathogens, little can be done to streamline BV treatment. Prior studies have been insufficient in comparing FRT bacteria on account of the limited number of species evaluated within a consistent model [15], [20]. Furthermore, variations between these studies make it impossible to compare host-bacterial interactions between reports. Here, we developed a coculture model to characterize the response of various FRT epithelial cells (the frontline in FRT mucosal defense) to a comprehensive collection of vaginal bacteria, including both commensal lactobacilli and BV-associated bacteria (BVAB). We evaluated a total of ten bacteria on three separate epithelial cell types, monitoring host response under consistent coculture conditions. In doing so, we observed distinct differences in immune responses between the three types of reproductive epithelia, as measured by cytokine and defensin induction. These responses demonstrated good agreement of our model with clinical BV samples. We also found that only a select few of the tested bacterial species elicited an immune response from host cells. Surprisingly, not all BVAB elicited potent immune responses, whereas one *Lactobacillus spp.* did stimulate significant cytokine and defensin induction in FRT epithelia. Thus, in addition to developing a model for immune interactions in the FRT, we also report unexpected trends in bacterial-host interactions, emphasizing the utility of this approach for understanding host-pathogen interactions in the FRT.

## Methods

### Reagents and Materials

Trizol and keratinocyte serum free media (KSFM) with supplements were from Invitrogen, while RNA Storage Solution and DNAse I kit were purchased from Ambion (both of Life Technologies, Carlsbad, CA, USA). Bio-Rad (Hercules, CA, USA) iScript and Sybr Green Supermix were used for RTqPCR experiments. RPMI1640, DPBS, and DMEM/F12 were from MediaTech, Inc., while collagen-coated Transwells were from Corning Life Sciences (both of Corning Inc, Corning, NY, USA). Fetal bovine serum (FBS) was from Gemini Bio-Products (West Sacramento, CA, USA).

### Epithelial and Bacterial Cultures

The following human epithelia were purchased from American Type Culture Collection (ATCC, Manassas, VA, USA): End1 (CRL-2615) from endocervix; Ect1 (CRL-2614) from ectocervix; VK2 (CRL-2616) from vagina. These were maintained according to ATCC instructions. Briefly, cells were grown in KSFM supplemented with additional calcium chloride, recombinant epidermal growth factor, and bovine pituitary extract. For maintenance, cultures were grown to 50–70% confluence before splitting. For Transwell experiments, cells were seeded at confluence (1.6×10^6^ End1 cells, 1.1×10^6^ Ect1 cells or 1.0×10^6^ VK2 cells, seeding varies according to cell kinetics). For all other experiments, cultures were grown to confluence on tissue culture-treated plates (Techno Plastic Products, Trasadingen, Switzerland). The following bacteria were purchased from ATCC as common representatives of commensal flora [21]–[29]: *Lactobacillus crispatus* (33197); *Lactobacillus acidophilus* (4356); *Lactobacillus johnsonii* (11506); *Lactobacillus jensenii* (25258); *Lactobacillus gasseri* (9857); *Lactobacillus vaginalis* (49540). The following bacteria were purchased from ATCC as representatives of BVAB [30], [31]: *Gardnerella vaginalis* (49145); *Atopobium vaginae* (BAA-55); *Mobiluncus curtisii* (35241); *Prevotella bivia* (29303). Lactobacilli were grown in MRS broth or plates at 37°C/5% CO_2_. *G. vaginalis*, *A. vaginae*, *M. curtisii*, and *P. bivia* were grown in tryptic soy broth (TSB) with 5% defibrinated rabbit blood (all media from Becton, Dickinson and Company, Franklin Lakes, NJ, USA), or on equivalent agar plates. *G. vaginalis* was grown at 37°C/5% CO_2_, while the other 3 BVAB were grown in anaerobic GasPaks (Becton, Dickinson and Company) at 37°C. To achieve consistency in bacterial preparations, maintenance cultures of each species were aliquoted and snap frozen by submerging in liquid nitrogen for 2 hr, then transferred to −80°C until use.

To prepare inocula for experiments, snap-frozen aliquots of aerobic bacteria were thawed and inoculated into prewarmed, pregassed MRS (for lactobacilli) or TSB (for *G. vaginalis*) for 2 hr to allow for recovery prior to coculturing. Desired volumes of cultures were then centrifuged at 4000×g for 10 min, supernatants were aspirated, and bacteria were resuspended in KSFM. Snap-frozen aliquots of anaerobic bacteria were thawed, and desired volumes were centrifuged. Supernatants were aspirated, and bacteria were resuspended in distilled water for one minute (to lyse erythrocytes carried over from maintenance media), and then diluted with four volumes KSFM (to restore osmolarity). This distilled water wash did not affect bacterial viability. The bacteria were centrifuged again, the supernatent was removed, and the bacteria were resuspended in KSFM.

In all experiments, the multiplicity of infection (MOI) was calculated as the number of bacterial colony forming units (CFUs) divided by the number of epithelial cells in a given coculture condition. The number of bacterial CFUs was determined by serially diluting the inocula and plating on appropriate media for back-calculation of inocula density. The final MOIs used were in agreement with reports of clinical bacterial load in the FRT, and with other published coculture models [20], [32], [33]. In bacterial-epithelial cocultures where bacterial stimulation of epithelia was compared, BVAB or *L. vaginalis* were applied at a lower MOI than nonstimulatory lactobacilli, to preclude concerns about small MOI differences effecting significant immune response differences. Coculture kinetics were monitored at the assay endpoint, and Supporting Information **Figures S1** and **S2** demonstrate bacterial and epithelial viability after coculture. Additionally, heat-killed inocula were evaluated for stimulatory BVAB to determine whether bacterial viability was required for epithelial stimulation. These data are presented in Supporting Information **Figure S3**.

### Bio-plex Analysis of Transwell Underlay

For Transwell cocultures, epithelial cells were seeded on 24 mm collagen-coated 0.4 µm Transwells. The next day, excess apical media and unattached cells were removed, basal media was changed, and cells were maintained at the air-liquid interface. Twenty-four hr after transitioning to air-liquid interface, underlay media was changed and cell monolayers were inoculated apically with 100 µL bacterial inoculum. Epithelia were coincubated with bacteria for 24 hr, then media underlay was collected and frozen at −20°C until analysis. For analysis, media underlay were clarified and analyzed by Bio-Rad Bio-plex multiplex cytokine array. Experimental analysis was performed according to manufacturer’s instructions.

### Bio-plex Analysis of Cervicovaginal Lavage Samples

CVLs were provided by HIV-negative participants in the Bronx/Manhattan consortium of the Women’s Interagency HIV Study (WIHS), a longitudinal observational cohort study of HIV-positive and HIV-negative women, at their routine semi-annual WIHS visits. Written informed consent was obtained from all participants, and samples were collected in accordance with protocols approved by the Institutional Review Board (IRB) of Montefiore Medical Center for this study. CVLs were obtained by irrigating the cervix and vaginal wall with 10 mL sterile saline as previously described [34] and cryopreserved at −80°C. BV was assessed by Amsel criteria [35] with the presence of at least three (of four) criteria indicating the presence of BV. For this study, the BV-negative group was comprised of samples that demonstrated the absence of all four criteria. Cervicovaginal lavage fluid was clarified before Bio-plex analysis. Experimental analysis was performed according to manufacturer’s instructions.

### Primary Lymphocyte Isolation

Venous blood was drawn from adult volunteers who provided written consent, and samples were obtained in accordance with a UCF IRB approved protocol for this study. Blood was drawn into acid citrate dextrose vacutainers (Becton, Dickinson and Company), and peripheral blood mononuclear cells (PBMCs) were separated within an hour of the donation. To separate PBMCs, whole blood was diluted in an equal volume of DPBS, manually overlaid on lymphocyte separation media (LSM, MP Biomedicals, Santa Ana, CA, USA), then centrifuged at 400×g for 30 min. PBMCs were isolated from LSM density gradients, and were washed twice with DPBS, then resuspended in RPMI containing 10% FBS and plated on tissue culture-treated plates (Techno Plastic Products). Plates were incubated at 37°C/5% CO_2_ for 2 hr before isolating non-adherent lymphocytes from adherent monocytes. Lymphocytes were maintained in RPMI with 10% FBS, and used within 24 hr of isolation.

### Chemotaxis of Primary Lymphocytes

Peripheral blood lymphocytes were resuspended in RPMI supplemented with 1% FBS at a density of two million cells per mL. Serial dilutions of recombinant human β-defensin 2 (hBD2, Peprotech, Rocky Hill, NJ, USA) or equivalent vehicle control were prepared in the same media. hBD2 and vehicle dilutions were plated in a ChemoTX plate (Neuroprobe, Gaithersburg, MD, USA), alongside media alone controls. The ChemoTX filter was attached to the plate, and 50 µL lymphocyte suspension was applied to the surface of each well. The ChemoTX plate was incubated at 37°C/5% CO_2_ for 3 hr. To compare migrated cells, media above the filter was removed, and apical surface of filter was washed once in DPBS with 5 mM EDTA, then incubated with the same wash for 30 min at 4°C. This second wash was removed, the ChemoTX plate was centrifuged at 400×g for 5 min, and the filter was removed. Cells in the lower chamber were resuspended in a volume of 100 µL, and the CytoTox Glo assay (Promega, Fitchburg, WI, USA) was used to compare total cell number according to manufacturer’s instructions. A standard curve of known cell numbers was used to calculate the number of migrated cells from relative light units.

### hBD2 Acid-Urea (AU) Western

Epithelial cells were harvested and lysed by scraping into 10% acetic acid. These cell lysates were vortexed 30 min at room temperature to extract protein. Soluble extracts were clarified and concentrated, then resolved on an acid-urea polyacrylamide gel electrophoresis (AU-PAGE). A standard of recombinant hBD2 was run alongside cell extracts on each gel. Gels were transferred to PVDF membranes and blotted with a goat polyclonal antibody against hBD2 (Peprotech).

### ELISA

Cell culture supernatants from treated epithelial cells were clarified and subjected to ELISA quantification using the Peprotech hBD2 ELISA Development Kit and Becton, Dickinson and Company OptEIA IL-6 and IL-8 Kits. Assays were performed according to suppliers’ instructions.

### Real-Time Quantitative Polymerase Chain Reaction (RTqPCR)

To isolate RNA, epithelial cells were rinsed in cold DPBS, harvested in Trizol reagent, and stored at −80°C until extraction. RNA was then precipitated, treated with DNAse I, and reverse transcribed. cDNAs were analyzed by RTqPCR using the following primer pairs: hBD2_F atctcctcttctcgttcctcttc; hBD2_R ccacaggtgccaatttgtttatac; hBD3_F cttctgtttgctttgctcttcc; hBD3_R cacttgccgatctgttcctc; GAPDH_F tggtatcgtggaaggactc; GAPDH_R agtagaggcagggatgatg. All cycle thresholds were averaged from duplicate reactions. Cycle thresholds for hBD amplicons were normalized to the GAPDH standard, and fold expression was calculated using the delta-deltaCt method. All fold expressions were reported as increases compared to a 0 hr control.

### Statistical Analyses

All statistical analyses were carried out in Microsoft Excel or GraphPad Prism. For Bio-plex cytokine data, raw values were log-transformed and two-way ANOVA with Bonferroni post test was used to compare bacterial and mock conditions from three independent experiments. For RTqPCR data, fold expression values for each condition were calculated relative to 0 hr. Fold expression of mock versus treated conditions was compared using one-way ANOVA for endpoint analysis, or two-way ANOVA for timecourse analysis, with Bonferroni post test. For chemotaxis experiments, cell numbers were normalized to media-only (control treatment) wells, and increase in hBD2 over matched vehicle wells was compared by two-way ANOVA with Bonferroni post tests. For IL-6, IL-8 and hBD2 ELISAs, fold expression was calculated compared to mock-treated cells, and one-way ANOVA with Tukey-Kramer post test was performed to compare *L. vaginalis* to other lactobacilli, or to BVAB. For figures in which only two conditions are directly compared, Student’s t-test was used.

## Results

### BV-Associated Bacteria Induce a Cytokine Response From Reproductive Epithelial Cells

**Figure 1:**
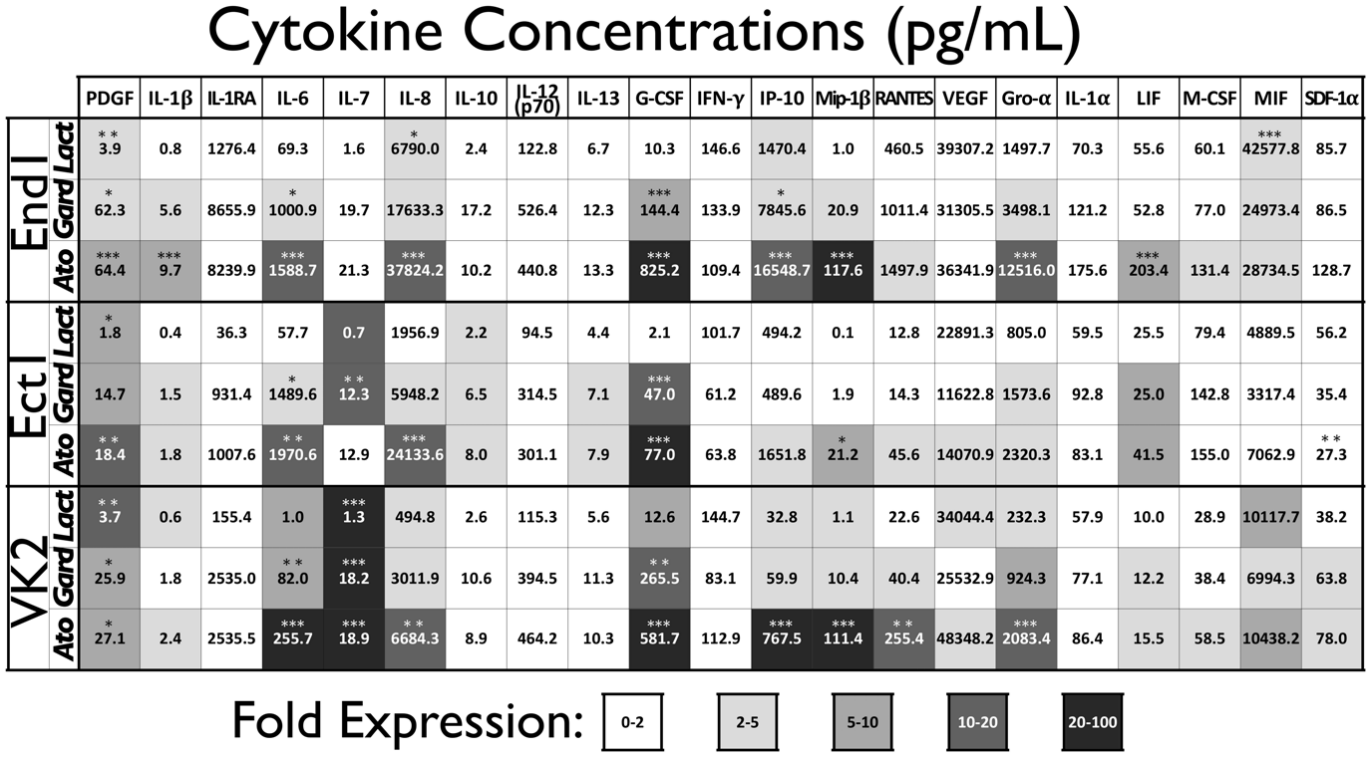
BVAB Induce an Innate Cytokine Response in Female Reproductive Epithelia. Transwell monolayers of each epithelial line were inoculated with indicated bacteria (Lact = *L. johnsonii*, average MOI = 33, Gard = *G. vaginalis*, average MOI = 15, Ato = *A. vaginae,* average MOI = 13) and 24 hr post-inoculation conditioned media were analyzed by multiplex cytokine bead array. Cytokine values are averaged from three independent experiments. Relative induction, represented here as fold expression and shaded accordingly, was calculated as the average increase in cytokine concentration compared to mock-inoculated control cells from three independent experiments. Significant differences in cytokine concentrations between bacteria-inoculated and mock condition are indicated by one (p<0.05), two (p<0.01) or three (p<0.001) asterisks.

The pathogenic sequelae resulting from BV may be attributed to the host inflammatory response to pathogenic BV-associated bacteria (BVAB). We hypothesized that this inflammatory response is mediated by the epithelial cells that line FRT and represent the initial point of contact for bacteria invading the FRT. In order to measure the contribution of FRT epithelial cells to BV-associated inflammation, we developed a coculture model to measure host epithelial response to BVAB. Epithelial cells derived from the vagina (VK2), ectocervix (Ect1), or endocervix (End1) have been previously used as a model to evaluate FRT microbicide tolerance and immune response [15], [20]. In our initial characterization, we sought to identify the cytokine repertoire that these epithelia are capable of producing in response to bacterial stimulation. Epithelia were seeded on transwells and inoculated at the air-liquid interface with a high MOI of either commensal bacteria (*Lactobacillus johnsonii*) or BVAB (*Gardnerella vaginalis* or *Atopobium vaginae*). After a 24 hr coculture, media underlay were analyzed in order to obtain a cytokine response profile for each epithelial type. Many of the analytes quantified were below the limit of detection (IL-2, IL2-Rα, IL-3, IL-4, IL-5, IL-9, IL-12p40, IL-15, IL-16, IL-17, IL-18, eotaxin, IFN-α2, FGF-β, GM-CSF, MCP-1, MCP-3, MIG, Mip-1α, SCF, SCGF-β, TNF-α, TNF-β, TRAIL, and HGF) and were not analyzed further. Others cytokines were below 50 pg/mL in all conditions (IL-1β, IL-7, IL-10, and IL-13), suggesting that the epithelia analyzed do not produce considerable concentrations of these cytokines under basal conditions or as part of their immune response to BVAB (**Figure 1**). On the other hand, the analytes IL-1RA, IL-6, IL-8, IP-10, RANTES, VEGF, Gro-α and MIF were recovered in nanogram/mL concentrations from some conditions, indicating that these cytokines are primary components of baseline or stimulated epithelial cytokine production. We compared the concentrations of these analytes in *L. johnsonii* condition versus BVAB conditions, and found that overall the BVAB induced greater cytokine responses from FRT epithelium than the commensal lactobacillus strain. The analytes IL-6, IL-8, G-CSF, IP-10, Mip-1β, RANTES, and Gro-α were upregulated in all three epithelial lines by BVAB more than by commensal lactobacilli, as indicated by the fold expression shading. Furthermore, the BVAB *A. vaginae* elicited more robust cytokine responses from each epithelial cell type compared to the other bacteria tested, with some chemokines achieving nanogram/mL concentrations in *A. vaginae-*stimulated conditions. Of note, we also observed greater cytokine production from End1 epithelial cells compared to the other two reproductive cell types.

**Figure 2:**
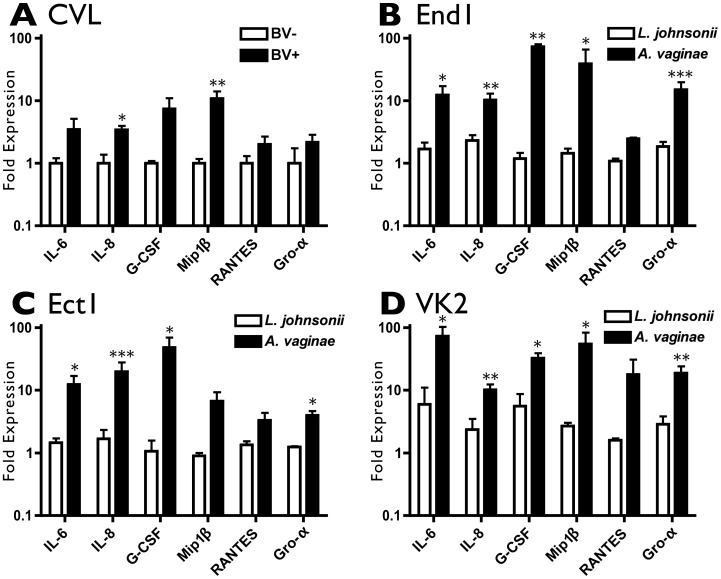
Epithelial Coculture Mirrors In Vivo Cytokine Response. A) Cervicovaginal lavage samples from BV-negative (n = 5) or BV-positive (n = 13) women were analyzed by multiplex cytokine bead array. Fold expression for each cytokine was calculated relative to the average value of the BV-negative samples. One (p<0.05) or two (p<0.01) asterisks indicate a significant increase in cytokine concentration for the BV-positive samples over the BV-negative samples. B-D) Cytokine induction in each epithelial line B) End1, C) Ect1, and D) VK2 in response to *L. johnsonii* (average MOI = 33) and *A. vaginae* (average MOI = 13) normalized to their paired mock-inoculated conditions. One (p<0.05), two (p<0.01), or three (p<0.001) asterisks indicate a significant increase in cytokine concentration for the *A. vaginae*-inoculated conditions over the *L. johnsonii*-inoculated conditions.

To demonstrate the relevance of our epithelial-bacteria coculture model, we compared the cytokine responses we observed in our epithelial model of BV with the cytokine trends of cervicovaginal lavage (CVL) samples obtained from a representative cohort of women with or without BV. Previous studies have reported increased concentrations of IL-1β, IL-6 and IL-8 in BV-positive CVL compared to BV-negative samples [9], [10], [11], [12]. Here, we use a small cohort exhibiting trends consistent with the literature for comparison to our model. The cytokine concentrations in CVL were quantified, and fold increases in each cytokine between BV-positive and BV-negative women were compared to the cytokine regulation between *A. vaginae*- and *L. johnsonii*-inoculated conditions in our FRT model (**Figure 2**). Shown are six cytokines, IL-6, IL-8, G-CSF, Mip-1 β, RANTES and Gro-α, that are elevated in clinical BV samples and similarly increased in our coculture model after inoculation with *A. vaginae*, but not with the commensal lactobacillus species *L. johnsonii*. Remaining cytokine data are including in Supporting Information **Figure S4**. Thus, our epithelial model aptly recapitulates the cytokine changes that occur in the reproductive tract as a result of BV. This comparison illustrates the contribution of FRT epithelia to the proinflammatory cytokine milieu that characterizes BV, and provides a convenient model for evaluating host-bacterial interactions in the reproductive tract. In addition to evaluating cytokines, we also investigated the induction of other innate immune effector proteins in response to BVAB.

### Reproductive Epithelia Upregulate hBD2, a Lymphocyte Chemoattractant, in Response to BVAB

**Figure 3:**
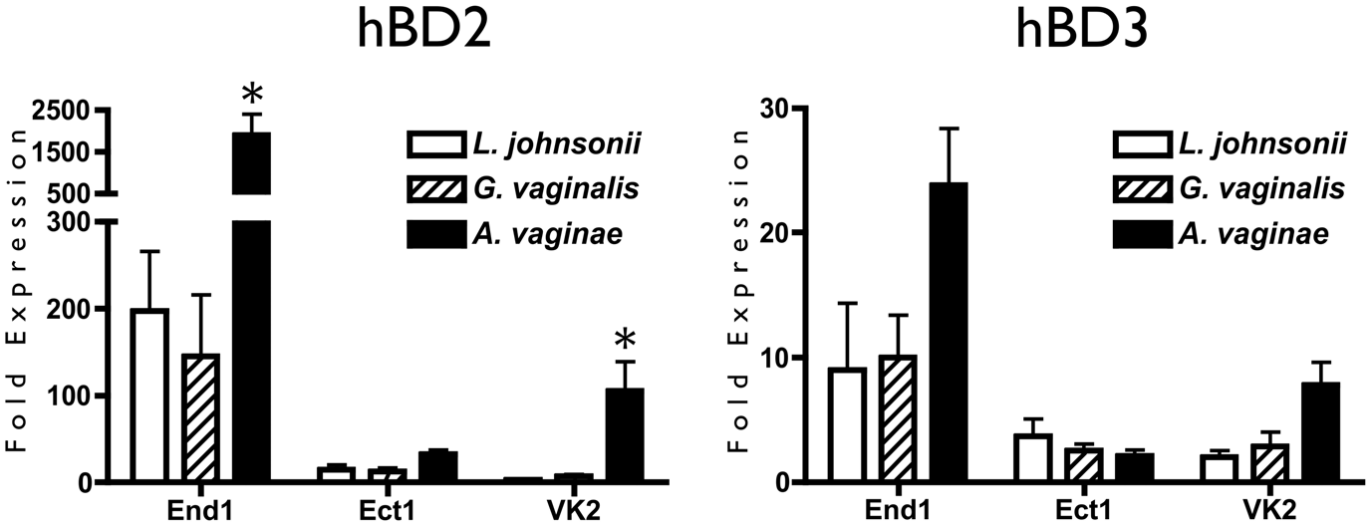
Human β-Defensin Gene Expression Is Upregulated in Reproductive Epithelia in Response to BVAB. Confluent monolayers of epithelial cells were inoculated with bacteria and coincubated for 48 hr, then analyzed by RTqPCR for hBD2 and hBD3 expression. Transcript expression is reported relative to 0 hr cells, and is shown as the average of three independent experiments. Average MOI are: 10 for *L. johnsonii*, 5.8 for *G. vaginalis*, and 5.6 for *A. vaginae*. Asterisks indicate a significant (p<0.05) increase over the *L. johnsonii*-treated condition.

Human defensins are important mediators of innate immunity, as they exhibit both antibacterial activity and chemotactic recruitment of immune cells, yet their role in BV has been studied with conflicting results [14], [15], [36]. To elucidate the host defensin response to BVAB, we measured hBD2 and hBD3 responses of reproductive epithelia at the transcript and protein levels following exposure to lactobacilli or BVAB. Epithelial cells were inoculated with commensal *L. johnsonii*, or pathogenic bacteria *G. vaginalis* or *A. vaginae,* and gene expression was measured by RTqPCR. In agreement with cytokine trends, hBD2 gene upregulation for each epithelial line was greatest in response to the pathogen *A. vaginae* (**Figure 3**). This upregulation was significant for End1 and VK2 cells, with End1 epithelia showed the greatest hBD2 response to bacterial stimulation compared to the other two epithelia. This is emphasized by a>1500-fold increase in hBD2 gene expression by End1 cells after inoculation with the BVAB *A. vaginae*. End1 and VK2 cells exhibited trends of hBD3 upregulation in response to *A. vaginae*, whereas the Ect1 cell type did not respond, indicating a difference in immune response to BVAB between cell types of the FRT.

**Figure 4:**
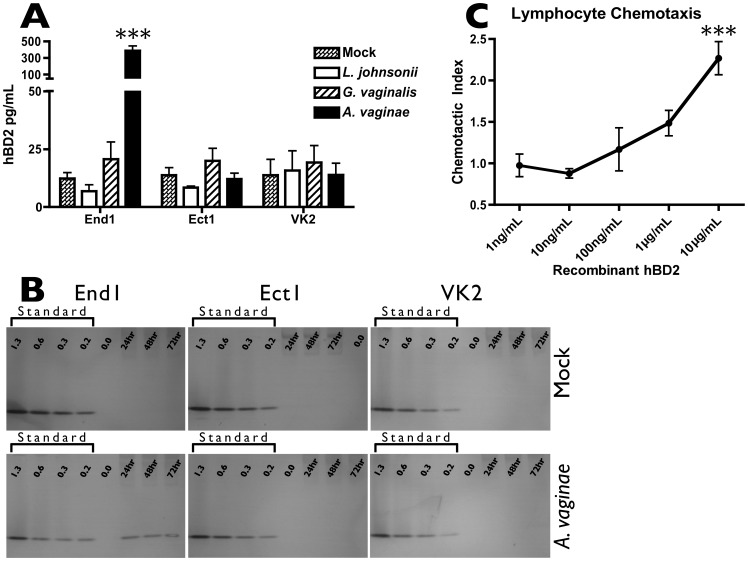
*A. vaginae* Induces Epithelial Expression of Soluble and Cell-Associated hBD2, a Protein That Attracts Primary Lymphocytes. A) Confluent monolayers of epithelial cells were inoculated with bacteria. Twenty-four hr post-inoculation, conditioned media were collected, clarified, and analyzed by ELISA to quantify concentrations of soluble hBD2 protein. Average MOI are: 5.8 for *L. johnsonii*, 2.5 for *G. vaginalis*, and 1.4 for *A. vaginae*. Results are averaged from 3 independent experiments, and asterisks indicate significant (p<0.001) increase over mock*-*treated condition. B) Confluent monolayers were inoculated with *A. vaginae*, or mock-inoculated. 24, 48, and 72 hr post-inoculation, cell monolayers were acid-extracted and analyzed by AU-PAGE western to quantify cell-associated hBD2 protein. A recombinant hBD2 protein standard (shown in ng per lane) was run alongside cell extracts for semi-quantitative comparison. Average MOI is 21. Shown is one example of three independent experiments. C) Unstimulated primary lymphocytes were isolated and evaluated for chemotaxis toward recombinant hBD2 protein. Chemotactic index is the ratio of migrated cells in hBD2-containing wells over vehicle-control wells, and significant increases over the matched vehicle condition are shown by three (p<0.001) asterisks.

To confirm the functional production of hBD2, we performed an ELISA to measure soluble hBD2 protein secreted by epithelia in response to bacterial exposure. hBD2 was quantified in conditioned media from epithelial cells cocultured with either commensal or pathogenic bacteria. In correspondence with transcript regulation, inoculation with BVAB resulted in a more robust upregulation of hBD2 protein by End1 cells compared to inoculation with commensal bacteria (**Figure 4** ***A***). However, neither Ect1 nor VK2 cells secreted significantly more hBD2 in response to BVAB than mock-inoculation.

In addition to measuring soluble hBD2, we considered that ELISA might not account for additional protein that remained cell-associated. To address this possibility, we performed AU-PAGE western blot to probe for cell-associated hBD2 in epithelia stimulated with *A. vaginae*. hBD2 protein was detected in End1 cell extracts after stimulation with BVAB *A. vaginae*, while neither Ect1 nor VK2 showed similar cell-associated protein (**Figure 4** ***B***). The cell-associated hBD2 protein recovered from *A. vaginae*-inoculated End1 cells appeared consistent over the three-day timecourse, and based on the internal protein standard, was calculated to contribute an additional 1.6 ng per 100 mm dish, compared to the 2.0 ng soluble protein per dish as measured by ELISA.

Previous reports have demonstrated that hBD2 protein stimulates chemotaxis of memory T cells and dendritic cells through the chemokine receptor CCR6. Having seen that hBD2 was significantly upregulated by reproductive epithelia in response to pathogenic bacteria, we sought to verify the ability of this chemoattractant to recruit primary lymphocytes. In agreement with previous reports [36], we observed dose-dependent increases in cell migration of unstimulated primary lymphocytes toward a recombinant hBD2 protein gradient (**Figure 4** ***C***). Importantly, our observation of hBD2-mediated chemotaxis of peripheral lymphocytes suggests that these cells can be recruited to tissues expressing increased levels of hBD2.

Having determined that reproductive epithelia provide a relevant model for characterizing host-pathogen interactions in the reproductive tract, we next sought to employ our model to explore the host response to a variety of reproductive bacteria, both commensal and pathogenic in nature.

### *Lactobacillus Vaginalis* Induces an Innate Immune Response

**Figure 5:**
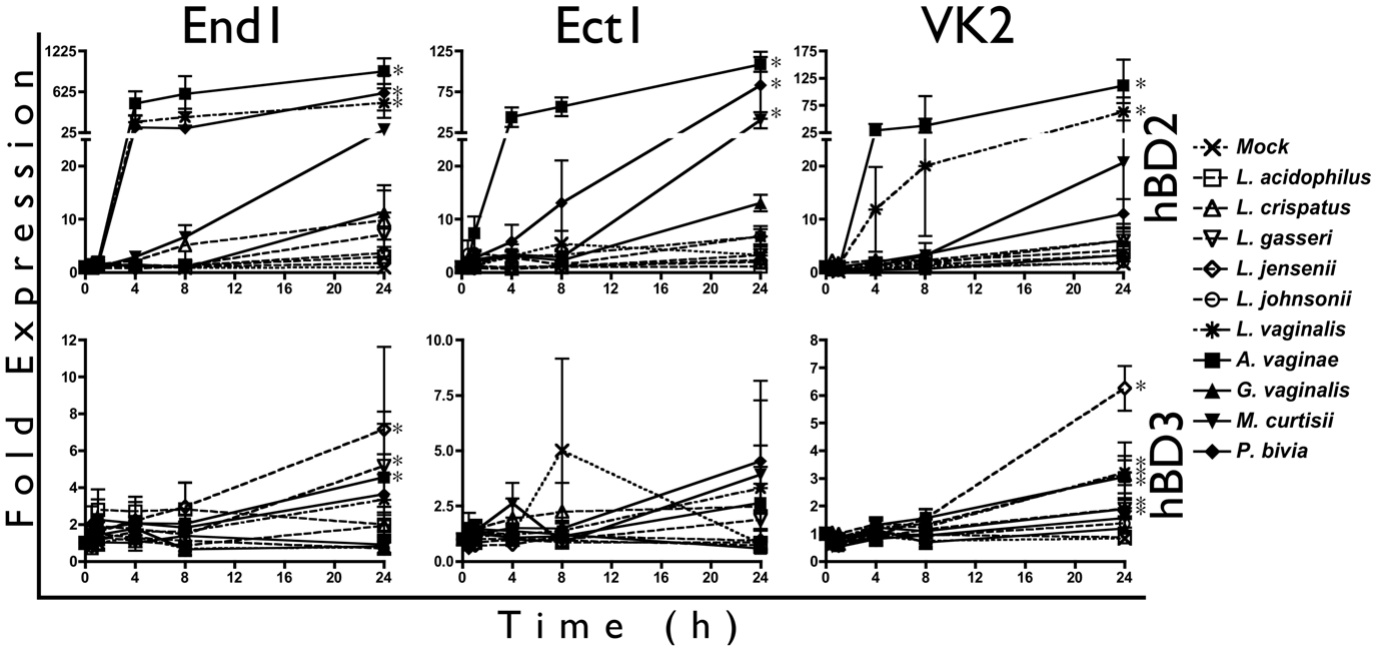
*L. vaginalis* Elicits hBD2 Gene Induction from Reproductive Epithelia. Confluent monolayers of epithelial cells were inoculated with bacteria and coincubated for up to 24 hr, then analyzed by RTqPCR for hBD2 and hBD3 expression. Transcript expression was normalized to mock-inoculated cells, and is shown as the average of three or four independent experiments. Average MOI are: 6.9 for *L. acidophilus,* 7.3 for *L. crispatus,* 9.7 for *L. gasseri,* 7.3 for *L. jensenii,* 7.3 for *L. johnsonii,* 3.4 for *L. vaginalis,* 3.7 for *A. vaginae,* 3.1 for *G. vaginalis,* 3.6 for *M. curtisii,* and 3.9 for *P. bivia*. Asterisks indicate a significant (p<0.05) increase in expression over mock-treated cells for at least one timepoint.

Since epithelial hBD2 response mirrored cytokine induction, we first used hBD gene expression as a predictive gauge of BVAB stimulatory capacity. We extended our analysis to a total of six lactobacillus strains (*Lactobacillus acidophilus, Lactobacillus crispatus, Lactobacillus gasseri, Lactobacillus jensenii, Lactobacillus johnsonii,* and *Lactobacillus vaginalis*) and four BVAB (*Atopobium vaginae, Garderella vaginalis, Mobiluncus curtisii* and *Prevotella bivia*). In agreement with our previous analyses, we observed that *A. vaginae* induced the most robust hBD2 gene upregulation (>100-fold increase in all three cell types), with *M. curtisii* and *P. bivia* eliciting considerable responses as well (**Figure 5**). Surprisingly, the lactobacillus species *L. vaginalis* also induced significant hBD2 gene upregulation from End1 (>400-fold) and VK2 cells (>60-fold), in contrast to its perceived commensal classification.

**Figure 6:**
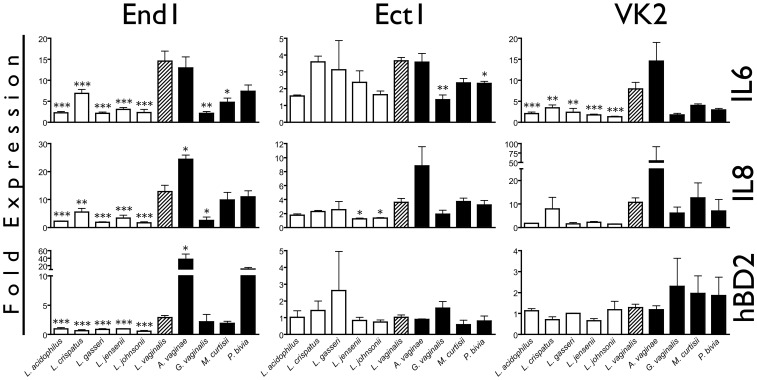
*L. vaginalis* Induces a Greater Immune Response than Other Vaginal Lactobacilli. Confluent monolayers of reproductive epithelia were inoculated with commensal lactobacilli or BVAB, and after 24 hr conditioned media was analyzed for IL-6, IL-8 and hBD2 protein. BVAB are filled black bars, *L. vaginalis* is hatched, and all other lactobacilli are white bars. MOI are the same as in Figure 5. Protein is shown as fold expression compared to a mock-treated condition, and one, two, or three asterisks indicate values that are significantly (p<0.05, p<0.01, or p<0.001, respectively) different from the *L. vaginalis*-treated condition.

To confirm the ability of *L. vaginalis* to induce an innate immune response similar to BVAB, we utilized ELISA to measure soluble IL-6, IL-8, and hBD2 proteins secreted by epithelia after bacterial inoculation. In comparison to the other five lactobacillus strains, *L. vaginalis* consistently induced a heightened response (**Figure 6**). In End1 cells, *L. vaginalis* stimulated>10-fold increase in both IL-6 and IL-8, which was significantly higher than all other lactobacilli tested. In agreement with our previous results, End1 cells were the only epithelia to produce considerable amounts of soluble hBD2 protein (picograms/mL) after coculture with bacteria, with *L. vaginalis* inducing significantly higher hBD2 levels than the other *Lactobacillus spp*.

**Figure 7:**
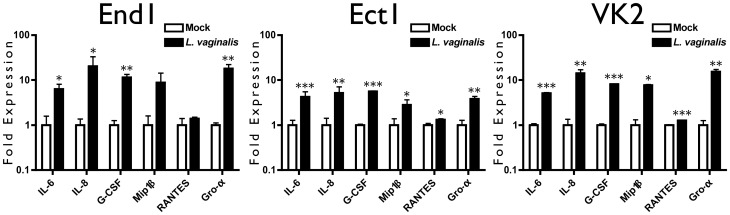
*L. vaginalis* Initiates an Innate Immune Response from FRT Epithelia. Confluent monolayers of epithelial cells were inoculated with bacteria and coincubated for 24 hr. Conditioned media were collected, clarified and ELISA was used to quantify hBD2, IL-6 and IL-8 concentrations. MOI is the same as in Figure Five. Analyte concentrations are shown as fold induction compared to mock condition, and are the average of three independent experiments where one (p<0.05), two (p<0.01), or three (p<0.001) asterisks indicate a significant increase over the mock-treated condition.

Finally, we performed Bio-Plex analysis to determine whether *L. vaginalis* promoted a cytokine environment similar to that observed clinically and in our coculture model of known BVAB (**Figure 7**). We observed that *L. vaginalis* stimulated significant upregulation of cytokines compared to mock-treated conditions. Analytes IL-6 and IL-8 were recovered at picogram/mL concentrations, corresponding to the magnitude recovered from BVAB cocultures. In agreement with other indicators, the cytokine trends elicited by *L. vaginalis* mirrored cytokine responses to BVAB and clinical BV trends, suggesting that this lactobacillus strain induces an innate immune response in reproductive epithelia.

## Discussion

In this study, we evaluated the immune response initiated by female reproductive epithelial cells in response to a comprehensive panel of ten bacteria, both commensal and pathogenic in nature, and showed that the overall cytokine response to bacterial pathogens aptly reflected the heightened cytokine environment that characterizes BV. Our side-by-side comparison of cytokine upregulation between our model and clinical BV samples demonstrated the appropriate recapitulation of physiological trends by our coculture method.

Like clinical CVLs, our epithelial coculture demonstrated upregulation of proinflammatory cytokines in response to pathogenic bacteria. While our model did reflect the heightened inflammatory environment created by IL-8, Gro-α and hBD2, other cytokines that are known to be upregulated in BV, including IL-1α and IL-1β [12], were upregulated in epithelia by stimulatory bacteria, but were not produced at appreciable levels. This is in agreement with the literature, which reports monocytic cell lineages as the major producers of IL-1 [37], thereby suggesting that other cell types in the reproductive tract likely contribute these factors to the BV milieu.

By using three different epithelial cell types, we demonstrated distinct differences in the immune responsiveness of epithelia along the reproductive tract. We consistently observed a heightened immune response from endocervical epithelia compared to ectocervical or vaginal cells. Our results suggest that the naturally colonized epithelia of the vagina and ectocervix display an attenuated immune response, perhaps in order to minimize excessive inflammatory recruitment triggered by transient changes in the dynamic microflora of the vagina. In line with this hypothesis, Ect1 and VK2 cells exhibited a markedly less robust hBD2 response to stimulatory bacteria (**Figures 3** and **4**). On the other hand, the endocervix acts as a transition zone to the sterile upper reproductive tract, and is not densely colonized by bacteria. Accordingly, bacterial contact results in a considerable increase in cytokine and defensin protein production; an appropriate response considering that pathogenic bacterial contact could threaten the sterility of the upper female reproductive tract [38], [39]. These findings demonstrate the utility of this model in characterizing epithelial function and behavior in the FRT.

In addition to evaluating three types of host epithelia, we also characterized relative stimulatory activity of ten FRT bacterial species, the most thorough comparison reported. We designed our cocultures so that BVAB were applied at a lower MOI than lactobacilli, and additionally found that they were recovered at lower density than lactobacilli at the end of the coculture. Still, BVAB induced an immune response greater than that induced by lactobacilli. It is likely that *in vivo* this difference in stimulatory activity is greater yet, considering that in BV pathogenic bacteria actively outgrow commensal lactobacilli. This implied difference in metabolic and proliferative capacity suggest that our report, though demonstrating considerable stimulation by BVAB, could be a conservative representation compared to *in vivo* stimulatory magnitude.

We observed that the BVAB, *A. vaginae,* induced the most robust response from all three epithelia as determined by cytokine and defensin upregulation. This is in concordance with recent research that shows *A. vaginae* as a more specific marker of clinical BV symptoms, and a stronger inducer of immune response than *G. vaginalis* [14], [40], [41]. In fact, while *G. vaginalis* was the first pathogen associated with BV [8], more thorough microbiome studies report the frequent isolation of *G. vaginalis* from BV-negative women [42], and in our analyses this bacterium induced responses similar in magnitude to the majority of commensal lactobacilli. This finding emphasizes the value of basic coculture studies to assist in identifying BVAB of clinical significance, and provides a framework in which additional strains may be evaluated for relative stimulatory activity.

When we expanded our analysis to characterize the β-defensin response of End1 cells, we observed significant upregulation of the inducible effector hBD2 that was both freely soluble and cell-associated. This is the first report to quantify cell-associated reservoirs of hBD2 in FRT epithelia, and these findings shed light on conflicting clinical data, which have reported both significant increases and significant decreases in hBD2 protein concentrations in the context of BV. Our data suggest that while hBD2 transcription is significantly upregulated in response to stimulatory bacteria, recovered soluble protein may not accurately depict this induction. *In vivo*, this may be partially due to dilution of total vaginal protein as a result of increased vaginal fluid discharge (a hallmark symptom of BV), but might also be attributed to retention of hBD2 protein as cell-associated protein. Furthermore, we demonstrated a considerable difference in the hBD2 protein production by different types of reproductive epithelia. In considering these factors, differences in sample method and sample site might contribute to the variation observed in hBD protein recovery from the FRT.

In total, we recovered on average 3.6 ng hBD2 per 100 mm dish, with 1.6 ng remaining cell-associated and 2 ng being freely secreted. The soluble portion of hBD2 alone is unlikely to reach antimicrobial concentrations (µg/mL levels) when secreted lumenally into the vaginal canal and diluted in vaginal fluid. However, it is likely that hBD2, secreted toward the basal submucosa, would reach chemotactic concentrations (30 ng/mL when the total protein is divided by cell monolayer volume). hBD2 is even more likely to achieve these levels at the basal cell surface if maintained in concentrated extracellular domains [43]. Extracellular stores of hBD2 may thus provide haptotactic stimuli for migrating lymphocytes, suggesting that hBD2 is not antibacterial, but rather chemotactic, in the setting of BV [44]. In line with our findings, an increased percent of CD4-positive lymphocytes has been reported in BV-positive vaginal fluid [45]. This may contribute to the increased HIV susceptibility that is associated with BV, as increased CD4-positive target cells concentrated in reproductive tissues may facilitate initial HIV infection. The chemotactic potential of defensin induction may represent an unfortunate host mediator of pathogenic processes.

Finally we used regulation of hBD2, IL-6 and IL-8 to evaluate the relative immune stimulation elicited by ten different vaginal bacteria. By all readouts, epithelial response to *L. vaginalis* was generally higher than the response to other lactobacilli. This was especially clear when analyzing the response from End1 epithelium, the most sensitive of the three cell types. The immune response elicited by *L. vaginalis* extended to the characteristic cytokine profile we observed for BVAB and for clinical CVL patterns, suggesting that this bacteria, unlike the other lactobacilli evaluated, induces an immune response from host cells that mimics a pathogen-triggered reaction. Clinical evaluation of *L. vaginalis* in reproductive afflictions is sparse, as few studies discern between different species of lactobacilli to obtain species-specific microbiome data [16], [17], [30]. However a recent study demonstrated that within a small sample group, *L. vaginalis* was cultured from 30% of ‘normal’ FRT individuals, and 50% of ‘disturbed’ FRT individuals (i.e. women with frequent BV-like vaginal microflora) [26], suggesting that this species may indeed play a role in FRT pathogenesis.

While the complexity of the FRT microbiome is just recently being appreciated, associations between individual bacteria and pathogenic sequelae remain uncharacterized. A recent report demonstrated associations between individual bacterial inhabitants and specific Amsel’s criteria [22]. Likewise, it stands to reason that the cytokine and defensin responses observed in BV are associated with certain specific bacterial subsets. Our results support this hypothesis, by demonstrating significant differences between the stimulatory capacities of individual BVAB. Combined with the growing appreciation of FRT microbiome diversity, our observations support reevaluation of FRT bacteria by coculture techniques in order to distinguish stimulatory bacterial strains from inert inhabitants.

## Supporting Information

Figure S1 **Bacterial Growth in Coculture is Minimal.** Confluent monolayers of epithelia or no epithelia control wells were inoculated with indicated bacteria as previously described. In addition to calculating starting inocula, we also monitored bacterial density at the experiment endpoint (24 hr) by resuspending the coculture and plating serial dilutions on appropriate bacterial growth media. Bacterial density is represented as back-calculated CFU, and is averaged from three independent experiments.
(TIF)

Figure S2 **Stimulatory BVAB do not Affect Epithelial Viability in Coculture.** Confluent monolayers of epithelia or no epithelia control wells were inoculated with indicated bacteria as described in Methods. At the coculture endpoint (24 hr) epithelial viability was assessed by CytoTox Glo system. Control wells without epithelia were subtracted from matched coculture conditions to account for background bacterial fluorescence. Percent viability is shown relative to mock-inoculated controls, and is averaged from three independent experiments. One or two asterisks indicate significant (p<0.05 and p<0.01, respectively) differences in viability compared to mock-inoculated controls.
(TIF)

Figure S3 **Heat-Killing of Bacterial Inocula Attenuates Epithelial Response.** Confluent monolayers of epithelia were inoculated with the BVAB *A. vaginae*, *M. curtisii* and *P. bivia* alongside heat-killed controls for each species. Heat-killing was achieved by incubating bacterial inocula at 65°C for 30 min, then cooling to 37°C prior to inoculation of epithelia, and was verified by plating. After 24 hr, epithelial response was measured by (A) IL-6 protein secretion (by ELISA), (B) IL-8 protein secretion (by ELISA), and (C) hBD2 transcript expression (by RTqPCR). All data are normalized to mock-inoculated controls and are averaged from three independent experiments. One or two asterisks indicate significant (p<0.05 and p<0.01, respectively) decrease in heat-killed condition compared to live bacterial inoculum.
(TIF)

Figure S4 **Supporting Bio-plex Cytokine Panel.** A) Analytes evaluated but not included in Figure 2 are provided for cervicovaginal lavage samples from BV-negative or BV-positive women, where fold expression for each cytokine was calculated relative to the average value of the BV-negative samples, and one (p<0.05), two (p<0.01), or three (p<0.001) asterisks indicate a significant increase for the BV-positive samples over the BV-negative samples. Of note, average values of IL-7 were 3.7 pg/mL for BV-negative group, and 8.5 pg/mL for BV-positive group. Averages for IL-1α were 616.6 pg/mL for BV-negative group, and 2455.1 pg/mL for BV-positive group. Averages for IL-1β were 165.5 pg/mL for BV-negative group, and 4924.4 pg/mL for BV-positive group. Also shown are cytokines for B) End1, C) Ect1, and D) VK2 in response to *L. johnsonii* and *A. vaginae* where one (p<0.05) or two (p<0.01) asterisks indicate a significant increase in cytokine concentration for the *A. vaginae*-inoculated conditions over the *L. johnsonii*-inoculated conditions. Refer to Figure 1 for average concentrations of each analyte in these conditions.
(TIF)
