# Supplementary figures and images for: Identification and Characterization of Bacterial Vaginosis-Associated Pathogens Using a Comprehensive Cervical-Vaginal Epithelial Coculture Assay
Source: PLoS One. 2012 Nov 15;7(11):e50106. doi: 10.1371/journal.pone.0050106 (PMC3499514; doi:10.1371/journal.pone.0050106)

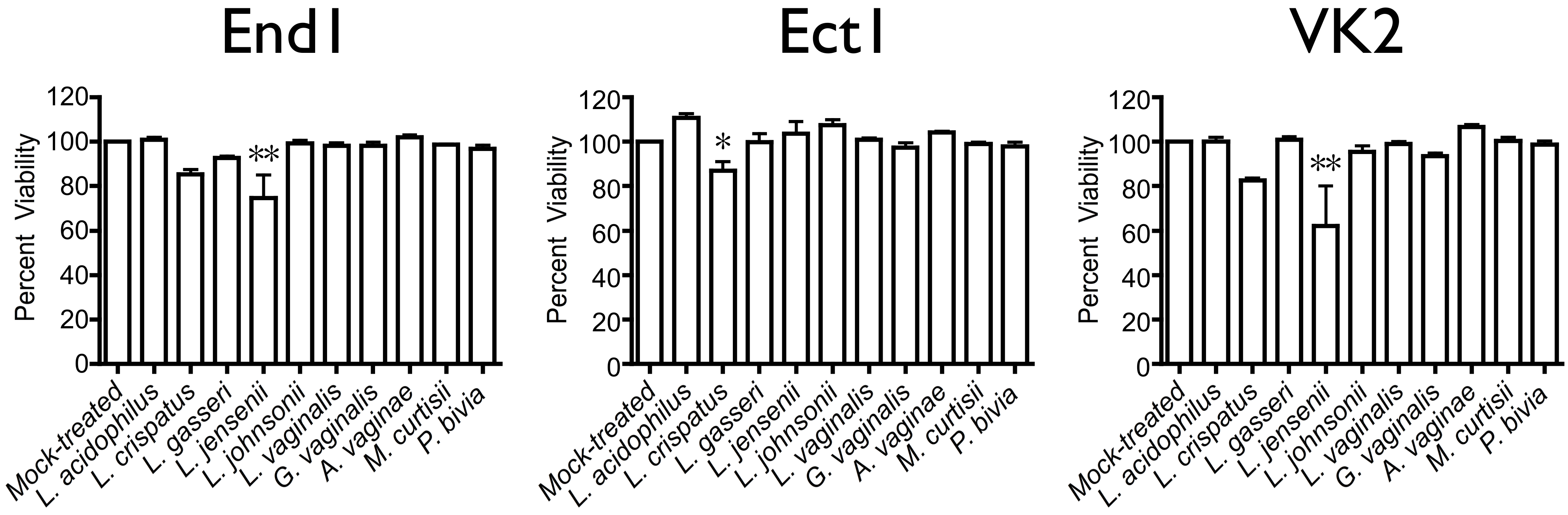

Supplement: Figure S2 — Stimulatory BVAB do not Affect Epithelial Viability in Coculture. Confluent monolayers of epithelia or no epithelia control wells were inoculated with indicated bacteria as described in Methods. At the coculture endpoint (24 hr) epithelial viability was assessed by CytoTox Glo system. Control wells without epithelia were subtracted from matched coculture conditions to account for background bacterial fluorescence. Percent viability is shown relative to mock-inoculated controls, and is averaged from three independent experiments. One or two asterisks indicate significant (p<0.05 and p<0.01, respectively) differences in viability compared to mock-inoculated controls. (TIF) [file pone.0050106.s002.tif]

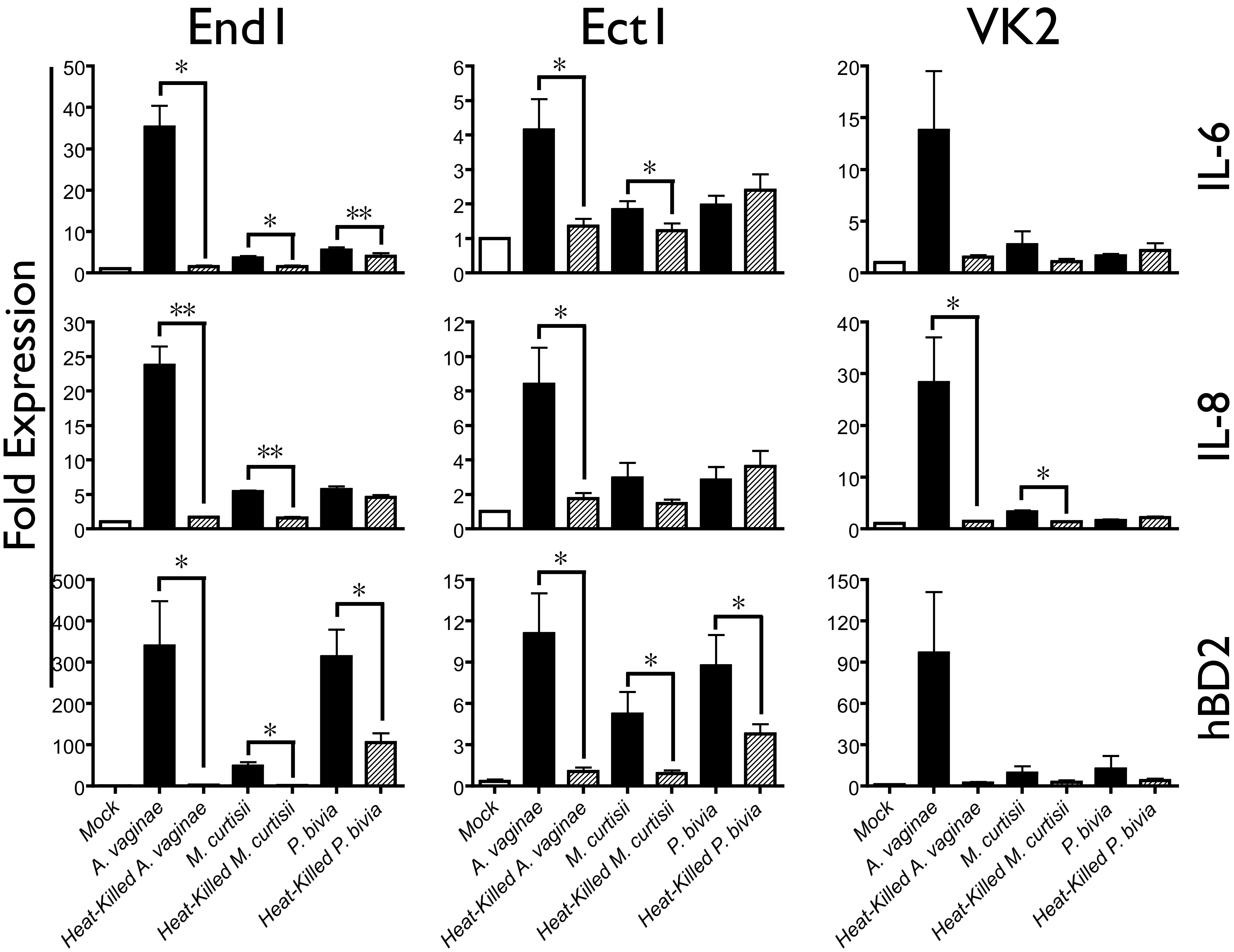

Supplement: Figure S3 — Heat-Killing of Bacterial Inocula Attenuates Epithelial Response. Confluent monolayers of epithelia were inoculated with the BVAB A. vaginae, M. curtisii and P. bivia alongside heat-killed controls for each species. Heat-killing was achieved by incubating bacterial inocula at 65°C for 30 min, then cooling to 37°C prior to inoculation of epithelia, and was verified by plating. After 24 hr, epithelial response was measured by (A) IL-6 protein secretion (by ELISA), (B) IL-8 protein secretion (by ELISA), and (C) hBD2 transcript expression (by RTqPCR). All data are normalized to mock-inoculated controls and are averaged from three independent experiments. One or two asterisks indicate significant (p<0.05 and p<0.01, respectively) decrease in heat-killed condition compared to live bacterial inoculum. (TIF) [file pone.0050106.s003.tif]

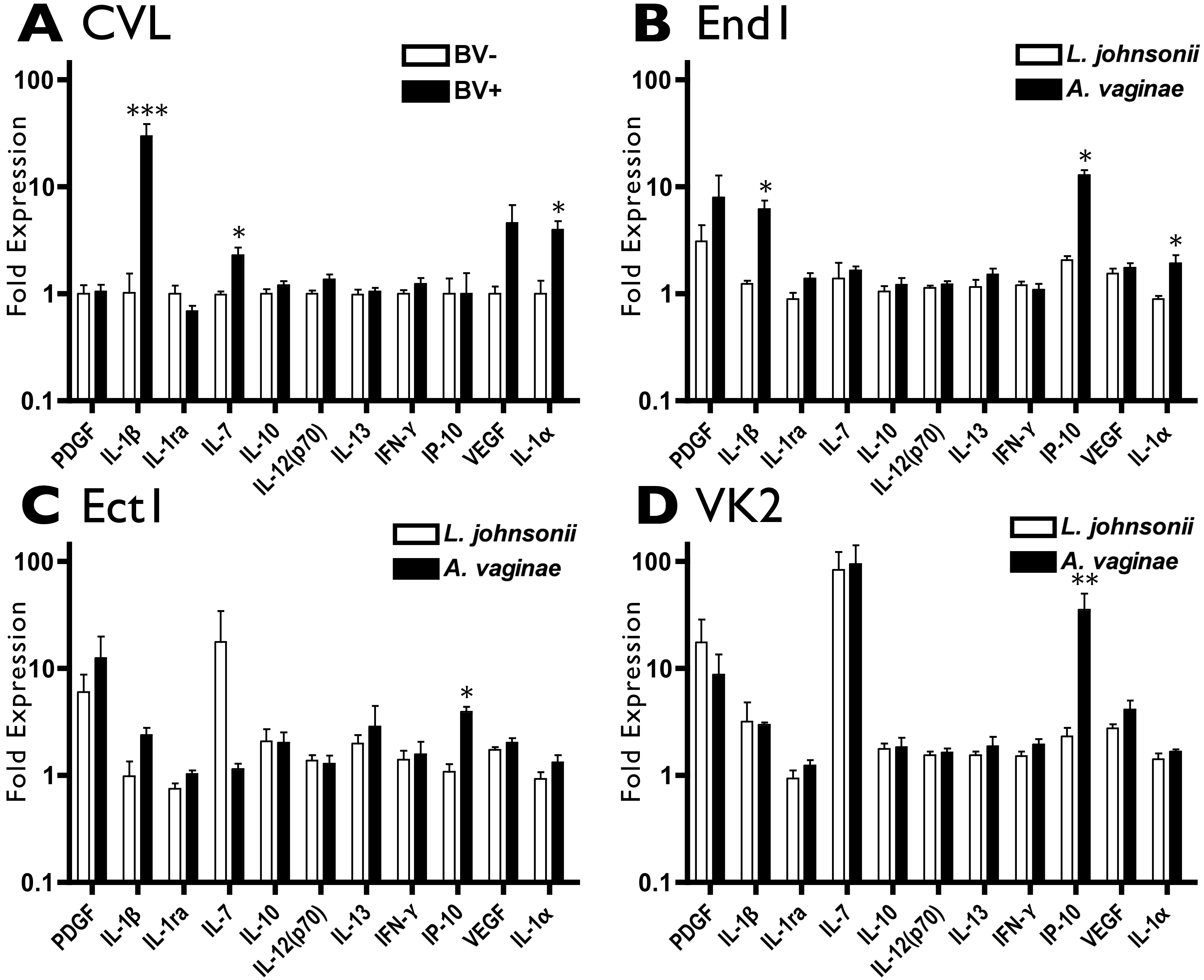

Supplement: Figure S4 — Supporting Bio-plex Cytokine Panel. A) Analytes evaluated but not included in Figure 2 are provided for cervicovaginal lavage samples from BV-negative or BV-positive women, where fold expression for each cytokine was calculated relative to the average value of the BV-negative samples, and one (p<0.05), two (p<0.01), or three (p<0.001) asterisks indicate a significant increase for the BV-positive samples over the BV-negative samples. Of note, average values of IL-7 were 3.7 pg/mL for BV-negative group, and 8.5 pg/mL for BV-positive group. Averages for IL-1α were 616.6 pg/mL for BV-negative group, and 2455.1 pg/mL for BV-positive group. Averages for IL-1β were 165.5 pg/mL for BV-negative group, and 4924.4 pg/mL for BV-positive group. Also shown are cytokines for B) End1, C) Ect1, and D) VK2 in response to L. johnsonii and A. vaginae where one (p<0.05) or two (p<0.01) asterisks indicate a significant increase in cytokine concentration for the A. vaginae-inoculated conditions over the L. johnsonii-inoculated conditions. Refer to Figure 1 for average concentrations of each analyte in these conditions. (TIF) [file pone.0050106.s004.tif]
